# Study of the photon’s pole structure in the noncommutative Schwinger model

**DOI:** 10.1140/epjc/s10052-014-2921-4

**Published:** 2014-06-12

**Authors:** M. Ghasemkhani

**Affiliations:** 1 Department of Physics, Shahid Beheshti University, G. C., Evin, Tehran, 19839 Iran; 2 School of Physics, Institute for Research in Fundamental Sciences (IPM), P.O.Box 19395-5531, Tehran, Iran

## Abstract

The photon self-energy of the noncommutative Schwinger model at two- and three-loop order is analyzed. It is shown that the mass spectrum of the model does not receive any correction from the noncommutativity parameter ($\theta$) at these orders. Also it remains unchanged to all orders. The exact one-loop effective action for the photon is also calculated.

## Introduction

The idea of noncommutative quantum field theory originates from the 1940s, when it was applied to cure the divergencies in quantum field theory before the renormalization approach was born [1]. It was demonstrated that the divergencies were not removed [2]. Later on, it was shown in [3] that the noncommutative quantum field theory describes effectively the low energy limit of the string theory on a noncommutative manifold. In the simplest case, the description of the noncommutative space-time is given by a constant parameter, $\theta ^{\mu \nu }$, of which the space-space (-time) components correspond to the magnetic (electric) field. The space-time noncommutative field theories suffer from the unitarity violation of the S-matrix [4] while the space-space noncommutative field theories face another obstacle, mixing of ultraviolet and infrared singularities [5]. The problem of the non-unitary S-matrix was studied in [6–8] but these works include some inconsistencies.

In fact, space-time noncommutativity leads to the higher orders of time derivatives of the fields in the Lagrangian which make the quantization procedure of the theory different from that of the commutative counterpart. For example in [9], the perturbative quantization of the noncommutative QED in $1+1$ dimensions has been analyzed up to ${\mathcal {O}}(\theta ^{3})$.

In the present work, the noncommutative two-dimensional QED with massless fermions in Euclidean space $(x_{2}\equiv it)$ is considered. The purpose of this paper is to concentrate on the mass spectrum of the theory at higher loops. The commutative counterpart of this model, the Schwinger model, was studied in [10] where it was shown that the photon in two dimensions acquires dynamical mass, arising from the loop effect, without gauge symmetry breaking. The mass spectrum of the Schwinger model contains a free boson with a mass proportional to the dimensionful coupling constant. Fermions disappear from the physical states due to the linearity of the potential that is similar to the quark confinement potential in quantum chromodynamics (QCD). Hence, the Schwinger model can be a toy model to understand the quark confinement. The extension of the Schwinger model to the noncommutative version as regards different aspects has been addressed in [9, 11–16]. Here, we focus on the dynamical mass generation in the noncommutative space.

This paper is organized as follows: in Sect. 2, we introduce the noncommutative Schwinger model in the light-cone coordinates in order to simplify our calculations. In Sect. 3, to obtain the mass spectrum of the theory at two- and three-loop order, the photon self-energy is studied. Using the explicit representation of the Dirac $\gamma$-matrices provides a straightforward method to compute the trace of the complicated fermionic loops. Then it is shown that the noncommutativity does not affect the Schwinger mass at these levels. The computations of Sect. 3 are extended to all orders in Sect. 4 where the exact mass spectrum is also obtained. In Sect. 5, we demonstrate that the noncommutative one-loop effective action for the photon is exactly the same as the commutative counterpart. Finally, Sect. 6 is devoted to the concluding remarks.

## Noncommutative Schwinger model in the light-cone coordinates

The Lagrangian of the noncommutative Schwinger model can be obtained from its commutative counterpart by replacing the ordinary product with the star-product, which is defined as follows:

2.1 $$\begin{aligned} f(x)\star g(x)\equiv \exp \left( \frac{i\theta _{\mu \nu }}{2}\frac{\partial }{\partial a_{\mu }}\frac{\partial }{\partial b_{\nu }}\right) f(x+a)g(x+b)\Bigg |_{a=b=0},\nonumber \\ \end{aligned}$$

where $\theta _{\mu \nu }$ is an antisymmetric constant matrix related to the noncommutative structure of the space-time. In two-dimensional space-time, $\theta _{\mu \nu }$ can be written as the antisymmetric tensor $\epsilon _{\mu \nu }$, which preserves the Lorentz symmetry, namely

2.2 $$\begin{aligned}{}[x_{\mu },x_{\nu }]=\theta \epsilon _{\mu \nu }. \end{aligned}$$

To avoid the unitarity problem in the noncommutative space-time field theories, we use the Euclidean signature throughout this paper. The Lagrangian of the two-dimensional noncommutative massless QED is given by

2.3 $$\begin{aligned} {\mathcal {L}}&= -i\bar{\psi }\star \gamma _{\mu }\partial ^{\mu } \psi +e \bar{\psi }\star \gamma _{\mu }A^{\mu }\star \psi +\frac{1}{4}F_{\mu \nu }\star F^{\mu \nu }\nonumber \\&+\frac{1}{2}(\partial _{\mu } A^{\mu })\star (\partial _{\nu } A^{\nu })\nonumber \\&-\partial _{\mu }\bar{c}\star (\partial ^{\mu }c-ie[A^{\mu },c]_{\star }), \end{aligned}$$

where $F_{\mu \nu }$ is defined as

2.4 $$\begin{aligned} F_{\mu \nu }= \partial _{\mu } A_{\nu }-\partial _{\nu } A_{\mu }+ie [A_{\mu },A_{\nu }]_{\star }, \end{aligned}$$

with $[A_{\mu },A_{\nu }]_{\star } = A_{\mu }\star A_{\nu }-A_{\nu }\star A_{\mu }$. One of the useful properties of the two-dimensional space is that our calculations in the light-cone coordinates, $x_{\pm }=x_{1}\pm ix_{2}$, are simplified significantly. The Lagrangian (2.3) in the light-cone gauge, $A_{-}=0$, has the following form:

2.5 $$\begin{aligned} {\mathcal L }&= -\frac{i}{2}\bar{\psi }\star (\gamma _{+}\partial _{-}+\gamma _{-}\partial _{+})\psi +\frac{e}{2} \bar{\psi }\star \gamma _{-} A_{+}\star \psi \nonumber \\&+\frac{1}{2}(\partial _{-}A_{+})\star (\partial _{-}A_{+}), \end{aligned}$$

where $$\gamma _{\pm }=\gamma _{1}\pm i\gamma _{2}$$ and $$A_{\pm }=A_{1}\pm iA_{2}$$.

**Fig. 1:**
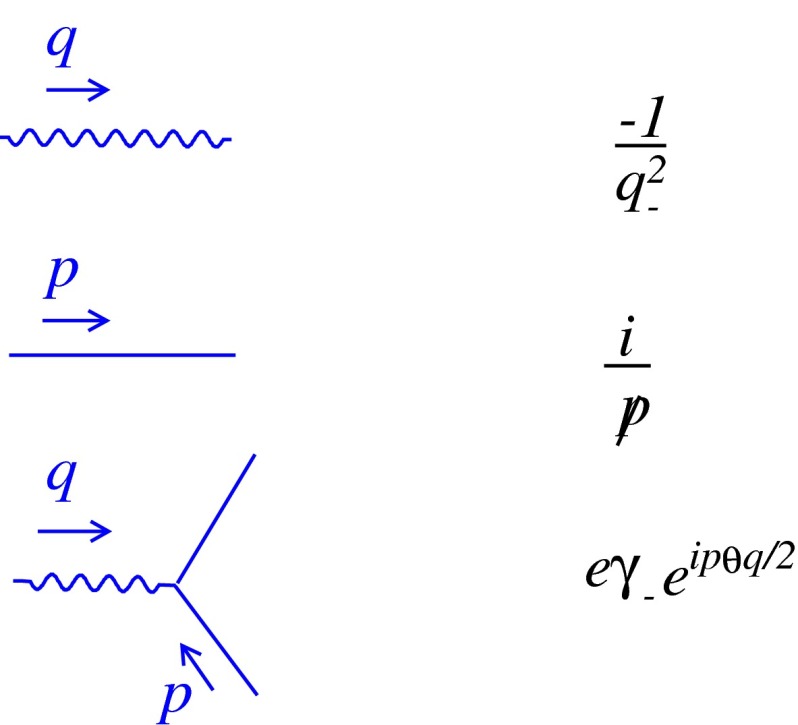
Feynman rules for noncommutative Schwinger model in the light-cone gauge

In this particular gauge, the non-linear term in the field strength tensor is removed. Therefore, the photon self-interaction parts, three- and four-photon interaction vertices, are eliminated and the ghost fields are decoupled from the theory. The resulting Feynman rules are shown in Fig. 1.

Note that only $\gamma _{-}$ appears in the fermion–photon vertex.

## Two- and three-loop noncommutative correction to the Schwinger mass

As was mentioned before, Schwinger showed that the photon in two dimensions acquires dynamical mass, $\mu =\frac{e}{\sqrt{\pi }}$. This mass generation originates from the presence of a special singularity in the scalar vacuum polarization at one-loop order. Using the non-perturbative method shows that the obtained mass does not receive any correction from loops at higher orders [10, 17]. The noncommutative extension of this kind of mass generation at one-loop level was discussed in [14] where it was proved that the Schwinger mass gets no noncommutative correction in this order. Higher-loop contributions, e.g. two- and three-loop contributions, have been pointed out in [15] without explicit computation of the loop integrals.

At two-loop order, there is only one diagram with $\theta$-dependent phase factor, but the three-loop order includes three $\theta$-dependent graphs. It is shown that the two- and three-loop computations are very similar. However, the analysis of the relevant three-loop graphs is a bit more complicated than that of the two-loop graph.

The general structure of the exact photon propagator^1^ in two-dimensional noncommutative space is the same as its commutative counterpart [14], namely

3.1 $$\begin{aligned} D^{\mu \nu }(q)=-\frac{\delta ^{\mu \nu }}{q^{2}[1+\Pi (q^{2})]}, \end{aligned}$$

where the scalar vacuum polarization, $\Pi (q^{2})$, is related to its tensor form via the following:

3.2 $$\begin{aligned} \Pi ^{\mu \nu }=(q^{2}\delta ^{\mu \nu }-q^{\mu }q^{\nu })\Pi (q^{2}), \end{aligned}$$

where $\Pi (q^{2})$ includes the commutative and noncommutative parts. The pole structure is obtained from the following limit:

3.3 $$\begin{aligned} \lim \limits _{q^{2}\rightarrow 0}q^{2}\Pi (q^{2},e^{2},\theta )&= \lim \limits _{q^{2}\rightarrow 0}q^{2}\Pi _{\text{ c }}(q^{2},e^{2})\nonumber \\&+\lim \limits _{q^{2}\rightarrow 0}q^{2}\Pi _{\text{ nc }}(q^{2},e^{2},\theta ), \end{aligned}$$

with fixed $\theta$. The first term yields the exact commutative Schwinger mass with $\Pi (q^{2},e^{2})=\frac{e^{2}}{\pi q^{2}}$ and the second term gives the noncommutative corrections to it. In the present section, we concentrate on the analysis of the second term in (3.3) at two- and three-loop level.

### Two-loop noncommutative correction

**Fig. 2:**
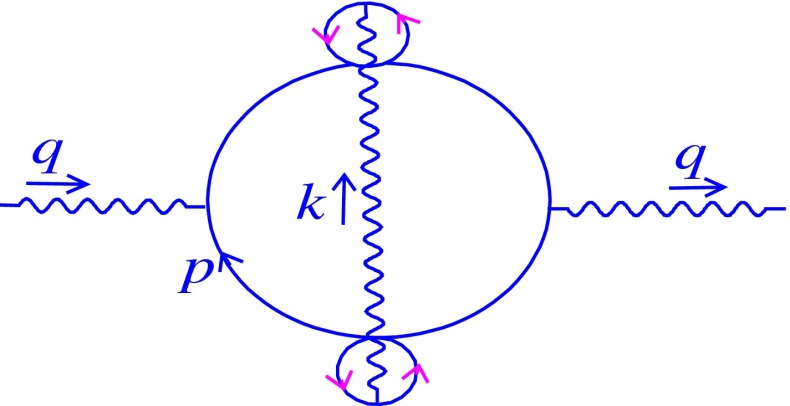
Relevant two-loop diagram

Two-loop order contains only one $\theta$-dependent diagram which is shown in Fig. 2. Here and in all figures of the paper, it is notable that a small circle oriented with pink arrows indicates a twist and does not show a fermionic loop. The Feynman form related to Fig. 2 is given by

3.4 $$\begin{aligned}&\Pi _{\mu \nu }^{(2)}|_{nc}=e^{4}\int \frac{d^{2}p}{(2\pi )^{2}}\frac{d^{2}k}{(2\pi )^{2}} \frac{1}{k^{2}} e^{-ik\theta q}\,tr\nonumber \\&\quad \times \bigg (\gamma _{\mu } \frac{1}{({\not }q+{\not }p)} \gamma ^{\rho }\frac{1}{({\not }q+{\not }p+{\not }k)} \gamma _{\nu }\frac{1}{({\not }p+{\not }k)}\gamma _{\rho } \frac{1}{{\not }p}\bigg ), \end{aligned}$$

which in the light-cone coordinates leads to the following:

3.5 $$\begin{aligned}&\Pi _{--}^{(2)}|_{nc}= e^{4}\nonumber \\&\quad \times \int \frac{dp_{-}dp_{+}}{(2\pi )^{2}}\frac{dk_{+}dk_{-}}{(2\pi )^{2}} \frac{g^{+-}e^{-ik\theta q}{\mathcal N}}{k^{2}_{-}(q+p)^{2}(q+p+k)^{2}(p+k)^{2}p^{2}},\nonumber \\ \end{aligned}$$

where

3.6 $$\begin{aligned} {\mathcal N}=tr(\gamma _{-} ({\not }q+{\not }p)\gamma _{-}({\not }q+ {\not }p+{\not }k) \gamma _{-}({\not }p+{\not }k)\gamma _{-} {\not }p ), \end{aligned}$$

and $k\theta q=\theta _{\mu \nu }k_{\mu }q_{\nu }=\frac{i\theta }{2}(k_{+}q_{-}-k_{-}q_{+})$. Using the explicit matrix form of $\gamma _{-}$ is useful to find the trace of the fermionic loop in a simple way (see Appendix A for more details). Therefore, the value of ${\mathcal N}$ is obtained as

3.7 $$\begin{aligned} {\mathcal N}= 2^{4}(p+q)_{-}(p+q+k)_{-}(p+k)_{-}p_{-}. \end{aligned}$$

Putting (3.7) in (3.5), we have

3.8 $$\begin{aligned}&\Pi _{--}^{(2)}|_{nc}=8 e^{4}\nonumber \\&\quad \times \int \frac{dp_{-}dp_{+}}{(2\pi )^{2}}\frac{dk_{+}dk_{-}}{(2\pi )^{2}} \frac{e^{-ik\theta q}(p+q)_{-}(p+q+k)_{-}(p+k)_{-}p_{-}}{k^{2}_{-}(q+p)^{2}(q+p+k)^{2}(p+k)^{2}p^{2}},\nonumber \\ \end{aligned}$$

that is rewritten as

3.9 $$\begin{aligned} \Pi _{--}^{(2)}|_{nc}= 8e^{4}\int \frac{dk_{+}}{2\pi }\frac{dk_{-}}{2\pi } \frac{1}{k_{-}^{2}} e^{-ik\theta q}{\mathcal E}, \end{aligned}$$

with

3.10 $$\begin{aligned} {\mathcal E}=\int \frac{dp_{-}}{2\pi }\frac{dp_{+}}{2\pi }\frac{1}{(p+q)_{+}(p+q+k)_{+} (p+k)_{+}p_{+}}.\nonumber \\ \end{aligned}$$

The produced phase factor in (3.9) is independent of the fermionic-loop momentum; hence the integral over $p$ can be evaluated separately.

To simplify (3.10), we decompose the fraction into partial fractions to reduce the degree of the denominator. The first step of the decomposition results in

3.11 $$\begin{aligned} {\mathcal E}&= \int \frac{dp_{-}}{2\pi }\frac{dp_{+}}{2\pi } \frac{1}{k^{2}_{+}}\bigg [\frac{1}{(p+q)_{+}}-\frac{1}{(p+q+k)_{+}}\bigg ]\nonumber \\&\times \bigg [\frac{1}{p_{+}}-\frac{1}{(p+k)_{+}}\bigg ]. \end{aligned}$$

Performing the complete decomposition produces the final expression as

3.12 $$\begin{aligned} {\mathcal E}&= \int \frac{dp_{-}}{2\pi }\frac{ dp_{+}}{2\pi } \frac{1}{k^{2}_{+}} \bigg \{ \frac{1}{q_{+}}\bigg [\frac{1}{p_{+}}-\frac{1}{(p+q)_{+}}\bigg ]\nonumber \\&-\frac{1}{(k-q)_{+}}\bigg [\frac{1}{(p+q)_{+}}-\frac{1}{(p+k)_{+}}\bigg ]\nonumber \\&-\frac{1}{(k+q)_{+}}\bigg [\frac{1}{p_{+}}-\frac{1}{(p+q+k)_{+}}\bigg ]\nonumber \\&+\frac{1}{q_{+}}\bigg [\frac{1}{(p+k)_{+}}-\frac{1}{(p+q+k)_{+}}\bigg ]\bigg \}. \end{aligned}$$

According to the complex form of Green’s theorem mentioned in [18], it is deduced that the $p$-integrals in each of the pairs separated in the parentheses vanish, namely ${\mathcal E}=0$. Hence

3.13 $$\begin{aligned} \Pi _{--}^{(2)}|_{nc}=0. \end{aligned}$$

If we use the electron mass as an infrared regulator, the obtained result remains unchanged. The detailed calculations with infrared regulator will be presented in Appendix B.

According to (3.3), the commutative Schwinger mass remains free from the noncommutative correction at two-loop order. In what follows, this calculation will be extended to three-loop level of the quantum corrections.

### Three-loop noncommutative correction

**Fig. 3:**
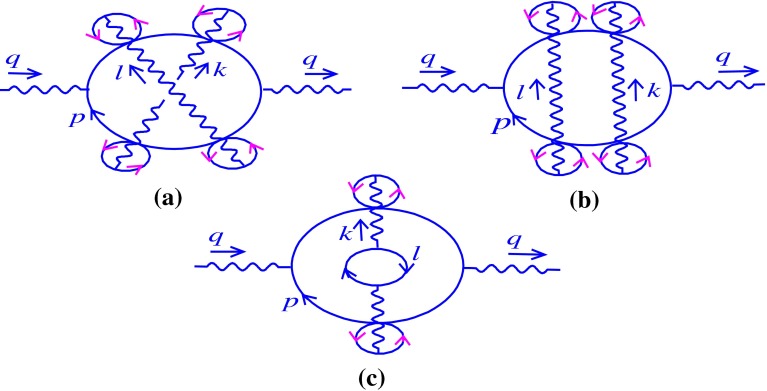
Some of the relevant three-loop diagrams

At three-loop order, unlike the two-loop case, there is more than one graph with $\theta$-dependent phase factor. Some of these graphs have been represented in Fig. 3. The contributions related to the graphs (a), (b), and (c) of Fig. 3 can be expressed as follows, respectively:3.14Here dots refer to the other diagrams that appear in this order. Rewriting (3.14) in the light-cone coordinates, we obtain3.15where3.16Having applied the relations mentioned in Appendix A, the explicit forms of the quantities ${\mathcal N}_{a}$ , ${\mathcal N}_{b}$, and ${\mathcal N}_{c}$ are given by

3.17 $$\begin{aligned} {\mathcal N}_{a}&= 2^{6} (p+q)_{-}(p+q+\ell )_{-}(p+q+\ell +k)_{-}\nonumber \\&\times (p+\ell +k)_{-}(p+k)_{-}p_{-},\nonumber \\ {\mathcal N}_{b}&= 2^{6} (p+q)_{-}(p+q+\ell )_{-}(p+q+\ell +k)_{-}\nonumber \\&\times (p+\ell +k)_{-}(p+\ell )_{-}p_{-},\nonumber \\ {\mathcal N}_{c}&= 2^{4}(p+q)_{-}(p+q+k)_{-}(p+k)_{-}p_{-}. \end{aligned}$$

Plugging them in (3.15), we have3.18As we see the phase factors appearing in (3.18), similar to the two-loop calculation, are independent of the fermionic-loop momentum. It can be shown that the other graphs, which appeared at three-loop level, also have a fermionic-loop momentum-independent noncommutative phase factor. In fact, this property remains true for all of the diagrams at any order [19]. Consequently, the $p$-integrals are calculated independently. Consider the first term of (3.18),

3.19 $$\begin{aligned} \Pi _{--}^{(3,a)}|_{nc}&= 16e^{6}\int \frac{dk_{+}dk_{-}}{(2\pi )^{2}}\frac{d\ell _{+}d\ell _{-}}{(2\pi )^{2}} \frac{1}{k_{-}^{2}\ell _{-}^{2}}\nonumber \\&\times e^{-i(k\theta \ell +k\theta q+\ell \theta q)}{\mathcal F}+\cdots , \end{aligned}$$

where

3.20 $$\begin{aligned}&{\mathcal F}=\int \frac{dp_{-}}{2\pi } \frac{dp_{+}}{2\pi }\nonumber \\&\quad \times \frac{1}{(p+q)_{+}(p+q+\ell )_{+}(p+q+\ell +k)_{+} (p+\ell +k)_{+}(p+k)_{+}p_{+}}.\nonumber \\ \end{aligned}$$

We use the decomposition method to simplify (3.20). Using the decomposition method at the first step leads to

3.21 $$\begin{aligned} {\mathcal F}&= \int \frac{dp_{-}}{2\pi }\frac{dp_{+}}{2\pi }\frac{1}{(kq\ell )_{+}}\nonumber \\&\times \bigg [\frac{1}{(p+q)_{+}}-\frac{1}{(p+q+\ell )_{+}}\bigg ]\nonumber \\&\times \bigg [\frac{1}{(p+k+\ell )_{+}}-\frac{1}{(p+q+\ell +k)_{+}}\bigg ]\nonumber \\&\times \bigg [\frac{1}{p_{+}}-\frac{1}{(p+k)_{+}}\bigg ], \end{aligned}$$

and in the second step, we find

3.22 $$\begin{aligned} {\mathcal F}&= \int \frac{dp_{-}}{2\pi }\frac{dp_{+}}{2\pi }\frac{1}{(kq\ell )_{+} }\bigg \{ \frac{1}{(\ell +k-q)_{+}}\nonumber \\&\times \bigg [\frac{1}{(p+q)_{+}}-\frac{1}{(p+q+k)_{+}}\bigg ]\nonumber \\&-\,\frac{1}{(\ell +k)_{+}}\bigg [\frac{1}{(p+q)_{+}}-\frac{1}{(p+q+\ell +k)_{+}}\bigg ]\nonumber \\&-\,\frac{1}{(k-q)_{+}}\bigg [\frac{1}{(p+q+\ell )_{+}}-\frac{1}{(p+\ell +k)_{+}}\bigg ]\nonumber \\&+\,\frac{1}{k_{+}}\bigg [\frac{1}{(p+q+\ell )_{+}}-\frac{1}{(p+q+\ell +k)_{+}}\bigg ] \bigg \}\nonumber \\&\times \bigg [\frac{1}{p_{+}}-\frac{1}{(p+k)_{+}}\bigg ]. \end{aligned}$$

After some algebraic manipulations, (3.22) is reduced to the following expression:

3.23 $$\begin{aligned} {\mathcal F}&= \int \frac{dp_{-}}{2\pi } \frac{dp_{+}}{2\pi }\bigg \{\frac{1}{(k q)_{+}}\frac{1}{(k+\ell )_{+}}\frac{1}{(q+\ell )_{+}}\frac{1}{(q+\ell +k)_{+}}\nonumber \\&\times \bigg [\frac{1}{p_{+}}-\frac{1}{(p+q+k+\ell )_{+}}\bigg ]\nonumber \\&+\,\frac{1}{(\ell q)_{+}}\frac{1}{(k+\ell )_{+}}\frac{1}{(k-q)_{+}}\frac{1}{(\ell +k-q)_{+}}\nonumber \\&\times \bigg [\frac{1}{(p+k+\ell )_{+}}-\frac{1}{(p+q)_{+}}\bigg ]\nonumber \\&+\,\frac{1}{(k\ell )_{+}}\frac{1}{(q+\ell )_{+}}\frac{1}{(k-q)_{+}}\frac{1}{(k-q-\ell )_{+}}\nonumber \\&\times \bigg [\frac{1}{(p+q+\ell )_{+}} -\frac{1}{(p+k)_{+}}\bigg ]\bigg \}. \end{aligned}$$

By a similar argument concerning (3.12), it is proved that ${\mathcal F}=0$. In the same way, the second and the third terms in (3.18) vanish. As a consequence

3.24 $$\begin{aligned} \Pi _{--}^{(3)}|_{nc}=0. \end{aligned}$$

In view of (3.3), it is deduced that the commutative Schwinger mass remains also untouched by noncommutativity at three-loop order. In the next section, this calculation will be extended to all orders.

## All-loop noncommutative correction to the Schwinger mass

**Fig. 4:**
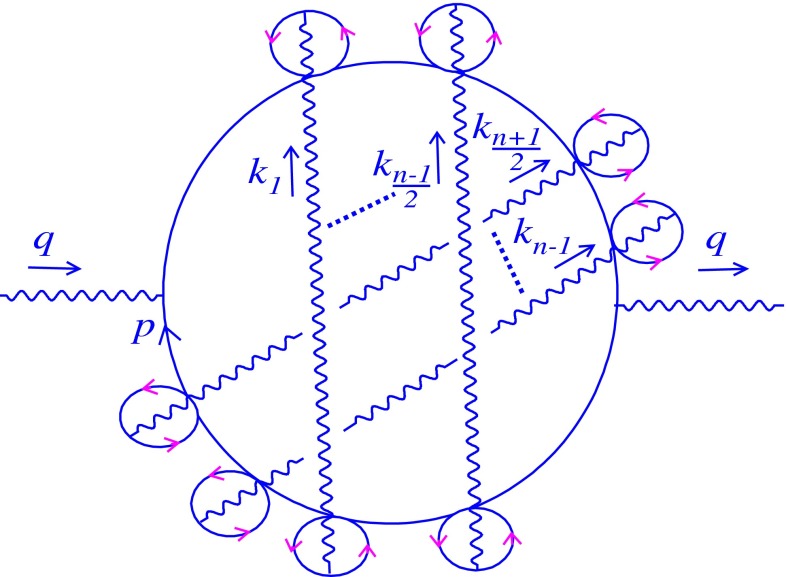
Relevant $n$-loop diagram

In this section, we generalize three-loop computation to all orders to obtain the exact mass spectrum. At $n$-loop level, there are several $\theta$-dependent diagrams contributing to the vacuum polarization tensor, and one of them may be found in Fig. 4, for which $n$ is an odd number.

The general Feynman form of Fig. 4 related to the photon’s vacuum polarization at $n$-loop ($n\ne 1$) is written as4.1where $\Pi _{--}^{(n,i)}|_{nc}$ shows the noncommutative contribution of the $i$th graph to the total self-energy at $n$-loop level. Analogous to Sect. 3, the numerator can easily be computed as

4.2 $$\begin{aligned} \Pi _{--}^{(n,i)}|_{nc}&= 2^{1-n} (e^{2})^{n}\int \frac{dp_{+}dp_{-}}{(2\pi )^{2}}\frac{dk_{1+}dk_{1-}}{(2\pi )^{2}}\frac{dk_{2+}dk_{2-}}{(2\pi )^{2}}\cdots \frac{dk_{(n-1)+}dk_{(n-1)-}}{(2\pi )^{2}} \frac{1}{k_{1-}^{2}k_{2-}^{2}\cdots k_{(n-1)-}^{2}}\nonumber \\&\times \exp \left[ i\left( q\theta \sum \limits _{r=1}^{n-1}k_{r} +\sum \limits _{r=1}^{\frac{n-1}{2}}k_{r}\theta \sum \limits _{s=\frac{n+1}{2}}^{n-1}k_{s}\right) \right] \nonumber \\&\times \frac{2^{2n} (q+p)_{-}(q+p+k_{1})_{-}\cdots \left( q+p+\sum \limits _{i=1}^{n-1}k_{i}\right) _{-} \left( p+\sum \limits _{i=1}^{n-1}k_{i}\right) _{-}\cdots p_{-}}{(q+p)^{2}(q+p+k_{1})^{2}\cdots \left( q+p+\sum \limits _{i=1}^{n-1}k_{i}\right) ^{2} \left( p+\sum \limits _{i=1}^{n-1}k_{i}\right) ^{2}\cdots p^{2}}. \end{aligned}$$

Due to the $p$-independence of the phase factor, (4.2) can be reduced to the following:

4.3 $$\begin{aligned}&\Pi _{--}^{(n,i)}|_{nc}= 2^{n+1}e^{2n}\int \frac{dk_{1+}dk_{1-}}{(2\pi )^{2}}\frac{dk_{2+}dk_{2-}}{(2\pi )^{2}}\cdots \nonumber \\&\quad \times \frac{dk_{(n-1)+}dk_{(n-1)-}}{(2\pi )^{2}} \frac{1}{k_{1-}^{2}k_{2-}^{2}\cdots k_{(n-1)-}^{2}}\nonumber \\&\quad \times \exp \left[ i\left( q\theta \sum \limits _{r=1}^{n-1}k_{r} +\sum \limits _{r=1}^{\frac{n-1}{2}}k_{r}\theta \sum \limits _{s=\frac{n+1}{2}}^{n-1}k_{s}\right) \right] {\mathcal G}, \end{aligned}$$

and $\mathcal {G}$ is defined as

4.4 $$\begin{aligned} {\mathcal G}=\int \frac{dp_{-}}{2\pi } \frac{dp_{+}}{2\pi } \frac{1}{(q+p)_{+}(q+p+k_{1})_{+}(p+q+k_{1}+k_{2})_{+}\cdots (q+p+\sum \limits _{i=1}^{n-1}k_{i})_{+} (p+\sum \limits _{i=1}^{n-1}k_{i})_{+}\cdots p_{+}}. \end{aligned}$$

It is proved that for a fixed $n$, similar to the previous section, the fraction in (4.4) can be decomposed into partial fractions such that it leads to ${\mathcal G}=0$. Thus

4.5 $$\begin{aligned} \Pi _{--}^{(n,i)}|_{nc}=0. \end{aligned}$$

The obtained result is correct for any $\theta$-dependent graph. Therefore, we conclude that

4.6 $$\begin{aligned} \sum \limits _{i}\Pi _{--}^{(n,i)}|_{nc}=0. \end{aligned}$$

Accordingly, the noncommutativity does not affect the Schwinger mass at all orders.

**Fig. 5:**
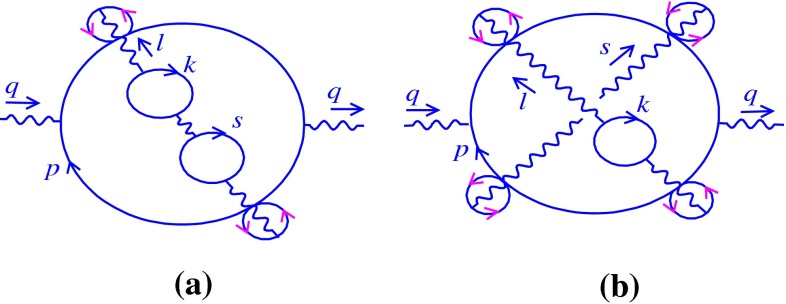
Relevant loop diagrams with internal fermionic-loop insertion

In particular, we note that diagrams like those shown in Fig. 5 with fermionic-loop insertion produce the noncommutative phase factors^2^ which are independent of the external fermionic-loop momentum. Hence, the evaluation of the integral over $p$ for these graphs will be similar to that of the graphs without the internal fermionic loops. Consequently, it is easily shown that the contribution of these graphs to the spectrum is also zero.

## Noncommutative one-loop effective action

**Fig. 6:**
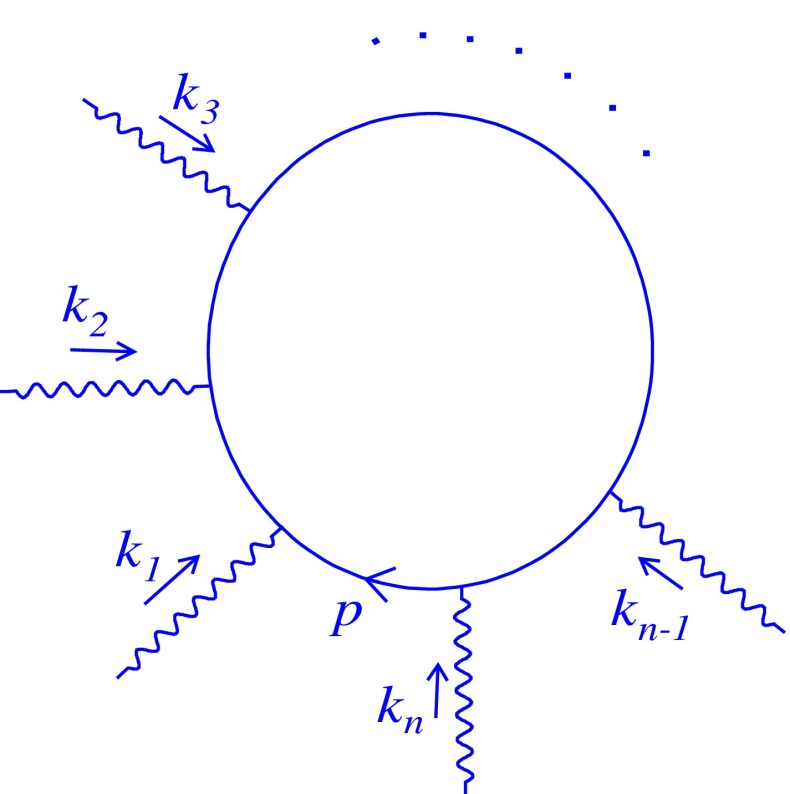
Relevant graph for the $n$th term of the one-loop effective action

The computation method used in two previous sections will be useful to simplify the photon’s one-loop effective action in the noncommutative space. The one-loop effective action in the commutative space, $\Gamma ^{c}[A]$, is given by integrating out the fermionic degrees of freedom,

5.1 $$\begin{aligned} \Gamma ^{c}[A]\equiv \int {\mathcal D}\bar{\psi }{\mathcal D}\psi \exp \bigg [i\int d^{2}x \bar{\psi }i{\not }D\psi \bigg ], \end{aligned}$$

where $D_{\mu }=\partial _{\mu }-ieA_{\mu }$ and $A_{\mu }$ is an external abelian gauge field. The quantity $\Gamma ^{c}[A]$ is equivalent to the following functional determinant from Fig. 6:5.2Using the non-perturbative approach in two dimensions, the expression $\Gamma [A]$ is exactly determined. In other words, (5.2) has a non-zero value only for $n=2$ which is equal to

5.3 $$\begin{aligned} \Gamma ^{c}[A]=-\frac{e^{2}}{2\pi }\int \frac{d^{2}k}{(2\pi )^{2}}A_{\mu }(k)\left( \delta ^{\mu \nu }-\frac{k^{\mu }k^{\nu }}{k^{2}}\right) A_{\nu }(-k).\nonumber \\ \end{aligned}$$

Therefore, the photon has received mass from the one-loop quantum correction.

The noncommutative version of $\Gamma [A]$ in three dimensions for non-abelian gauge fields has been already discussed in [20]. In what follows, we determine the one-loop effective action for the noncommutative Schwinger model. According to (5.1), we can define

5.4 $$\begin{aligned} \Gamma ^{nc}[A]\equiv \int {\mathcal D}\bar{\psi }{\mathcal D}\psi \exp \bigg [i\int d^{2}x \bar{\psi }\star i{\not }D\psi \bigg ], \end{aligned}$$

where $D_{\mu }=\partial _{\mu }-ieA_{\mu }\star$. Similar to the commutative part, $\Gamma ^{nc}[A]$ can be represented as5.5which is equivalent to the following expression:

5.6 $$\begin{aligned} \Gamma ^{nc}[A]&= \sum _{n=1}^{\infty }\int d^{2}z_{1}\cdots d^{2}z_{n} A^{\mu _{1}}(z_{1})\cdots A^{\mu _{n}}(z_{n})\nonumber \\&\times \Gamma _{\mu _{1}\cdots \mu _{n}}^{nc}(z_{1},\ldots ,z_{n}). \end{aligned}$$

The quantity $\Gamma _{\mu _{1}\cdots \mu _{n}}^{nc}(z_{1},\ldots ,z_{n})$ is given by^3^

5.7 $$\begin{aligned} \Gamma _{\mu _{1}\cdots \mu _{n}}^{nc}(z_{1},\ldots ,z_{n})&= \frac{(-e)^{n}}{n}\int \prod _{j=1}^{n}\frac{d^{2}k_{j}}{(2\pi )^{2}} (2\pi )^{2}\nonumber \\&\times \delta \left( \sum _{j=1}^{n}k_{j}\right) e^{i\sum \limits _{j=1}^{n}k_{j}z_{j}}e^{\frac{i}{2}\sum \limits _{j<\ell }^{n}k_{j}\theta k_{\ell }}\nonumber \\&\times \widetilde{\Gamma }_{\mu _{1}\cdots \mu _{n}}(k_{1},\ldots ,k_{n}), \end{aligned}$$

with5.8Since the noncommutative phase factor produced in (5.7), similar to Sects. 3 and 4, is also $p$-independent, the integral over $p$ can be separated from the rest, i.e. (5.8).

The non-zero leading term in (5.7) arises from $n=2$ which leads to its commutative value^4^, namely $\Gamma _{\mu _{1}\mu _{2}}^{nc}=\Gamma _{\mu _{1}\mu _{2}}^{c}$. For $n>2$, we just follow the technique applied for two- and three-loop calculations. Writing (5.8) in the light-cone coordinates, we arrive at5.9Using the detailed computations of Appendix A, (5.9) can be simplified as

5.10 $$\begin{aligned}&\widetilde{\Gamma }_{-\cdots -}(k_{1},\ldots ,k_{n})= \int \frac{dp_{-}}{(2\pi )}\frac{dp_{+}}{(2\pi )}\nonumber \\&\quad \times \frac{1}{(p+k_{1})_{+}(p+k_{1}+k_{2})_{+}(p+k_{1}+k_{2}+k_{3})_{+}\ldots p_{+}}.\nonumber \\ \end{aligned}$$

Analogous to (4.4), the relation (5.10) can be decomposed into partial fractions for a fixed $n$. After doing a complete decomposition and using the complex form of Green’s theorem, we obtain $\Gamma _{\mu _{1}\cdots \mu _{n}}^{nc}=0$ for $n>2$. Thus, the noncommutativity has no effect on the one-loop effective action and its exact commutative form is preserved. We have

5.11 $$\begin{aligned} \Gamma ^{nc}[A]=\Gamma ^{c}[A]&= -\frac{e^{2}}{2\pi }\int \frac{d^{2}k}{(2\pi )^{2}}A_{\mu }(k)\nonumber \\&\times \left( \delta ^{\mu \nu }-\frac{k^{\mu }k^{\nu }}{k^{2}}\right) A_{\nu }(-k). \end{aligned}$$

## Conclusion

In this paper, we have concentrated on the mass spectrum of the noncommutative Schwinger model with Euclidean signature at higher loops. It is demonstrated that the Schwinger mass receives no noncommutative corrections at all orders.

To prove this in a perturbative method, we have used the light-cone gauge to simplify the Lagrangian form. In this gauge, only the fermion–photon vertex remains and consequently the fermionic loops contribute to our calculations. Having fixed the gauge, the study of the noncommutative sector of the photon self-energy at two-, three-, and all-loop order has been performed.

At two- and three-loop level, the noncommutative parts of the photon self-energy were analyzed. Since the noncommutative phase factor appearing in the Feynman integrals is independent of the fermionic-loop momentum, the corresponding loop integral is easily evaluated. This analysis showed that the contributions from the $\theta$-dependent graphs are zero. Hence, the commutative mass spectrum does not change at these orders. Then the calculation of Sect. 3 was extended to all orders. Similar to two- and three-loop level, the noncommutative phase factor is independent of the fermionic-loop momentum and the resulting integral vanishes. This proves that the Schwinger mass remains intact at all orders in the noncommutative space.

The technique applied for computing the trace of the fermionic loops inspired us to study the relevant one-loop effective action. As a consequence, the exact one-loop effective action in the light-cone gauge with no noncommutative corrections was obtained.

Using the arguments of Sects. 3 and 4, it is possible to extend the analysis of the one-loop effective action to all loops. It is easily shown that the all-loop photon’s effective action, similar to the one-loop effective action, does not also receive noncommutative corrections. Although we have investigated in this paper only the photon sector, it would be interesting to do a similar analysis for the fermion self-energy and the running of coupling constant, in which case noncommutativity corrections are expected to appear.

## Appendix A: Two-loop fermionic trace in the light-cone coordinates

In this appendix, we present more details of the computation of the trace expression appearing in the relation (3.6).

A.1 $$\begin{aligned}&{\mathcal N}=tr\bigg (\gamma _{-} ({\not }q+{\not }p)\gamma _{-}({\not }q+ {\not }p+{\not }k)\gamma _{-}({\not }p+{\not }k) \gamma _{-} {\not }p \bigg ). \end{aligned}$$

To calculate this, we start from the representation of the gamma matrices in Euclidean space

A.2 $$\begin{aligned} \gamma _{1}=\left( \begin{array}{l@{\quad }l} 0 &{} -i \\ i &{} 0 \\ \end{array} \right) ,\quad \gamma _{2}=\left( \begin{array}{c@{\quad }c} 0 &{} -1 \\ -1 &{} 0 \\ \end{array}\right) , \end{aligned}$$

which in the light-cone coordinates are defined as

A.3 $$\begin{aligned} \gamma _{+}\!=\!\gamma _{1}+i\gamma _{2}\!=\! \left( \begin{array}{c@{\quad }c} 0 &{} -2i\\ 0 &{} 0 \\ \end{array}\right) ,\quad \gamma _{-}\!=\!\gamma _{1}-i\gamma _{2}= \left( \begin{array}{c@{\quad }c} 0 &{} 0\\ 2i &{} 0 \\ \end{array}\right) , \end{aligned}$$

and the light-cone metric by using $g^{\mu \nu }{=}\frac{\partial x^{\mu }}{\partial x^{\rho }}\frac{\partial x^{\nu }}{\partial x^{\sigma }}\delta ^{\rho \sigma }$ is obtained

A.4 $$\begin{aligned} g^{\mu \nu }=\left( \begin{array}{c@{\quad }c} g^{++} &{} g^{+-}\\ g^{-+} &{} g^{--} \\ \end{array}\right) = \left( \begin{array}{c@{\quad }c} 0 &{} \frac{1}{2}\\ \frac{1}{2} &{} 0 \\ \end{array}\right) . \end{aligned}$$

The terms such as ${\not }p$ appeared in (A.1) can be revised as follows:

A.5 $$\begin{aligned} {\not }p=\frac{1}{2}(p_{+}\gamma _{-}+p_{-}\gamma _{+})= \left( \begin{array}{c@{\quad }c} 0 &{} -ip_{-}\\ ip_{+} &{} 0 \\ \end{array}\right) , \end{aligned}$$

consequently

A.6 $$\begin{aligned} \gamma _{-}{\not }p=\left( \begin{array}{c@{\quad }c} 0 &{} 0\\ 2i &{} 0 \\ \end{array}\right) \left( \begin{array}{c@{\quad }c} 0 &{} -ip_{-}\\ ip_{+} &{} 0 \\ \end{array}\right) =\left( \begin{array}{c@{\quad }c} 0 &{} 0\\ 0&{} 2p_{-} \\ \end{array}\right) . \end{aligned}$$

Plugging (A.6) in (A.1) we have,

A.7 $$\begin{aligned} {\mathcal N}&=tr\bigg [ \left( \begin{array}{c@{\quad }c} 0 &{} 0\\ 0&{} 2(p_{-}+q_{-}) \\ \end{array}\right) \left( \begin{array}{c@{\quad }c} 0 &{} 0\\ 0&{} 2(p+q+k)_{-} \\ \end{array}\right) \\&\quad \times \left( \begin{array}{c@{\quad }c} 0 &{} 0\\ 0&{} 2(p+k)_{-} \\ \end{array}\right) \left( \begin{array}{c@{\quad }c} 0 &{} 0\\ 0&{} 2p_{-} \\ \end{array}\right) \bigg ] \\&=tr\left( \begin{array}{c@{\quad }c} 0 &{} 0\\ 0&{} 2^{4}(p+q)_{-}(p+q+k)_{-}(p+k)_{-}p_{-} \\ \end{array}\right) \\&=2^{4} (p+q)_{-}(p+q+k)_{-}(p+k)_{-}p_{-}. \end{aligned}$$

## Appendix B: Two-loop photon self-energy with mass insertion

Our purpose of the present appendix is to illustrate that the electron mass insertion as an infrared regulator does not change the result (3.13). To prove this, let us start from the relation (3.5) by rewriting it with the mass term

B.1 $$\begin{aligned}&\Pi _{--}^{(2)}|_{nc}= e^{4}\int \frac{dp_{-}dp_{+}}{(2\pi )^{2}}\frac{dk_{+}dk_{-}}{(2\pi )^{2}} g^{+-}e^{-ik\theta q}{\mathcal N}_{_{m}}\\&\quad \times \frac{1}{k^{2}_{-}[(q+p)^{2}-m^{2}][(q+p+k)^{2}-m^{2}][(p+k)^{2}-m^{2}][p^{2}-m^{2}]}, \end{aligned}$$

where

B.2 $$\begin{aligned} {\mathcal N}_{_{m}}&=tr\bigg (\gamma _{-} ({\not }q+{\not }p+m)\gamma _{-}({\not }q+ {\not }p+{\not }k+m)\gamma _{-}\\&\quad \times ({\not }p+{\not }k+m) \gamma _{-} ({\not }p+m) \bigg ). \end{aligned}$$

Similar to (A.5), the matrix form of ${\not }p+m$ in light-cone coordinates is given by

B.3 $$\begin{aligned} {\not }p+m= \left( \begin{array}{c@{\quad }c} m &{} -ip_{-}\\ ip_{+} &{} m \\ \end{array}\right) , \end{aligned}$$

and multiplying by $\gamma _{-}$ we have,

B.4 $$\begin{aligned} \gamma _{-}({\not }p+m)=\left( \begin{array}{c@{\quad }c} 0 &{} 0\\ 2i &{} 0 \\ \end{array}\right) \left( \begin{array}{c@{\quad }c} m &{} -ip_{-}\\ ip_{+} &{} m \\ \end{array}\right) =\left( \begin{array}{c@{\quad }c} 0 &{} 0\\ 2im&{} 2p_{-} \\ \end{array}\right) . \end{aligned}$$

Substituting (B.4) in (B.2) yields

B.5 $$\begin{aligned} {\mathcal N}_{_{m}}&=tr\bigg [ \left( \begin{array}{c@{\quad }c} 0 &{} 0\\ 2im&{} 2(p_{-}+q_{-}) \\ \end{array}\right) \left( \begin{array}{c@{\quad }c} 0 &{} 0\\ 2im&{} 2(p+q+k)_{-} \\ \end{array}\right) \\&\quad \times \left( \begin{array}{c@{\quad }c} 0 &{} 0\\ 2im&{} 2(p+k)_{-} \\ \end{array}\right) \left( \begin{array}{c@{\quad }c} 0 &{} 0\\ 2im&{} 2p_{-} \\ \end{array}\right) \bigg ]\\&=16 tr\left( \begin{array}{c@{\quad }c} 0 &{} 0\\ im {\mathcal J}&{} {\mathcal J}\\ \end{array}\right) =16{\mathcal J}, \end{aligned}$$

with

B.6 $$\begin{aligned} {\mathcal J}=(p+q)_{-}(p+q+k)_{-}(p+k)_{-}p_{-}. \end{aligned}$$

As we see, the mass of the electron does not appear in the final result of the trace expression. Hence, the relations (B.5) and (A.7), corresponding to ${\mathcal N}_{_{m}}$ and ${\mathcal N}$, respectively, are exactly the same, apart from a numerical factor.

Inserting (B.5) in (B.1) and simplifying, we arrive at

B.7 $$\begin{aligned} \Pi _{--}^{(2)}|_{nc}= 8e^{4}\int \frac{dk_{+}}{2\pi }\frac{dk_{-}}{2\pi } \frac{e^{-ik\theta q}}{k^{2}_{-}} {\mathcal E}_{_{m}}, \end{aligned}$$

where

B.8 $$\begin{aligned} {\mathcal E}_{_{m}}=\int \frac{dp_{-}}{2\pi }\frac{dp_{+}}{2\pi }\frac{1}{\left[ (p+q)_{+}-\frac{m^{2}}{(p+q)_{-}}\right] \left[ (p+q+k)_{+}-\frac{m^{2}}{(p+q+k)_{-}}\right] \left[ (p+k)_{+}-\frac{m^{2}}{(p+k)_{-}}\right] \left[ p_{+}-\frac{m^{2}}{p_{-}}\right] }. \end{aligned}$$

Decomposing the integrand of (B.8) into partial fractions, we have

B.9 $$\begin{aligned}&{\mathcal E}_{_{m}}=\int \frac{dp_{-}}{2\pi }\frac{dp_{+}}{2\pi } \left( \frac{1}{k_{+}+\frac{m^{2}k_{-}}{(p+q)_{-}(p+q+k)_{-}}}\right) \\&\quad \times \left( \frac{1}{(p+q)_{+}-\frac{m^{2}}{(p+q)_{-}}} -\frac{1}{(p+q+k)_{+}-\frac{m^{2}}{(p+q+k)_{-}}}\right) \\&\quad \times \left( \frac{1}{k_{+}+\frac{m^{2}k_{-}}{p_{-}(p+k)_{-}}}\right) \left( \frac{1}{p_{+}-\frac{m^{2}}{p_{-}}}-\frac{1}{(p+k)_{+}-\frac{m^{2}}{(p+k)_{-}}}\right) , \end{aligned}$$

which leads to the final result:

B.10 $$\begin{aligned} {\mathcal E}_{_{m}}&=\int \frac{dp_{-}}{2\pi }\frac{dp_{+}}{2\pi }\\&\times \left( \frac{1}{k_{+}+\frac{m^{2}k_{-}}{(p+q)_{-}(p+q+k)_{-}}}\right) \left( \frac{1}{k_{+}+\frac{m^{2}k_{-}}{p_{-}(p+k)_{-}}}\right) \\&\times \left\{ \left( \frac{1}{q_{+}+\frac{m^{2}q_{-}}{q_{-}(p+q)_{-}}}\right) \right. \\&\times \left. \left[ \frac{1}{p_{+}-\frac{m^{2}}{p_{-}}} -\frac{1}{(p+q)_{+}-\frac{m^{2}}{(p+q)_{-}}}\right] \right. \\&\left. -\left( \frac{1}{(k-q)_{+}+\frac{m^{2}(k-q)_{-}}{(p+q)_{-}(p+k)_{-}}}\right) \right. \\&\left. \times \left[ \frac{1}{(p+q)_{+}-\frac{m^{2}}{(p+q)_{-}}} -\frac{1}{(p+k)_{+}-\frac{m^{2}}{(p+k)_{-}}}\right] \right. \\&\left. -\left( \frac{1}{(k+q)_{+}+\frac{m^{2}(k+q)_{-}}{p_{-}(p+q+k)_{-}}}\right) \right. \\&\left. \times \left[ \frac{1}{p_{+}-\frac{m^{2}}{p_{-}}} -\frac{1}{(p+q+k)_{+}-\frac{m^{2}}{(p+q+k)_{-}}}\right] \right. \\&\left. +\left( \frac{1}{q_{+}+\frac{m^{2}q_{-}}{(p+k)_{-}(p+q+k)_{-}}}\right) \right. \\&\left. \times \left[ \frac{1}{(p+k)_{+}-\frac{m^{2}}{(p+k)_{-}}}-\frac{1}{(p+q+k)_{+}-\frac{m^{2}}{(p+q+k)_{-}}}\right] \right\} . \end{aligned}$$

Using the complex version of Green’s theorem yields ${\mathcal E}_{_{m}}=0$ and consequently $\Pi _{--}^{(2)}|_{nc}=0$.

## Footnotes

1 Here we work in Feynman gauge.

2 The noncommutative phase factors related to the graphs (a) and (b) in Fig. 5 are $e^{-i(\ell \theta q)}$ and $e^{-i(\ell \theta q+s\theta q+s\theta \ell )}$, respectively.

3 In the commutative case, $\theta =0$, only $\Gamma _{\mu _{1}\mu _{2}}^{c}$ has a non-zero value and $\Gamma _{\mu _{1}\cdots \mu _{n}}^{c}$ vanishes for $n>2$.

4 Since for $n=2$ the noncommutative phase factors arising from two vertices cancel each other, we obtain the commutative result (5.3).
